# The Comparison of Vaginal Cream of Mixing Yogurt, Honey and Clotrimazole on Symptoms of Vaginal Candidiasis

**DOI:** 10.5539/gjhs.v7n6p108

**Published:** 2015-04-03

**Authors:** Maryam Darvishi, Fereshteh Jahdi, Zeinab Hamzegardeshi, Saied Goodarzi, Mohsen Vahedi

**Affiliations:** 1 School of Nursing and Midwifery, Tehran University of Medical Sciences, Tehran, Iran; 2 Department of Midwifery, School of Nursing and Midwifery, Iran University of Medical Sciences, Tehran, Iran; 3 Department of Reproductive Health and Midwifery, Mazandaran University of Medical Sciences, Sari, Iran; 4 Traditional and Complementary Medicine Research Centre, Mazandaran University of Medical Sciences, Sari, Iran; 5 Faculty of Pharmacy, Tehran University of Medical Sciences, Tehran, Iran; 6 Department of Epidemiology andBiostatistics, School of Public Health, Tehran University of MedicalSciences, Tehran, Iran

**Keywords:** vulvovaginal candidiasis, honey, yogurt, clotrimazole

## Abstract

**Background:**

Vulvovaginal candidiasis is known as one of the most common fungal infection among women of reproductive age and considered as an important public health problem. In recent years, due to resistance to common antifungal medication, the use of traditional medicine of anti-fungal and herbal treatmentis increased. Therefore the objective of this study was to determine the effects of vaginal cream, mixture of yogurt and honey and comparing it with clotrimazole vaginal cream on symptoms of Vulvovaginal candidiasis in patients.

**Methods:**

In this randomized, triple blind clinical trial of 70 non-pregnant women infected with Candidalvulvovaginitis were placed in two groups of Vaginal cream mixed of yogurt and honey recipients (N = 35) and clotrimazole vaginal cream (N = 35). Both groups were treated for 7 days.At the beginning of study, Clinical and laboratory signs and symptoms were registered 7 and 14 days after treatment by questionnaire, observation formand secretions medium culture results. Data were analyzed by chi-square test, t test, McNemar tests through SPSS version 21. Significance level of 0.05 was considered.

**Results:**

The result of present study reveals the significant differences in symptom improvement of yogurt and honey, toward clotrimazole group (P < 0.05) and also Positive results of the first cultures (one week after treatment) in “yogurt and honey” and clotrimazole (20% versus 8.6%) and second time cultivation (14 days after treatment) (17.1% versus 8.6%) were similar and there was no significant differences between the two groups. (P > 0.05).

**Conclusion:**

This study indicated that the therapeutic effects of vaginal cream, yogurt and honey is not only similar with clotrimazole vaginal cream but is more effective in relieving some symptoms of vaginal candidiasis. Therefore, the use of this product can be suggested as an herbal remedy for candida infection treatment.

## 1. Introduction

Vulvovaginal candidiasis is one of the gynecological problems in womenand common fungal infection in adult women in reproductive age (Abdelmonem, Rasheed, & Mohamed, 2012). Around the world women with any races and at any ages would infected to Vulvovaginal candidiasis and this disease is one of the most repeated diseases (Babic & Hukic, 2010). This infection is responsible for about 13 million vaginitis in women in North America and 64 million women atreproductive age are affected in Brazil (Achkar & Fries, 2010) the prevalence of Candida in Iran have been reported 18/5-26% (Pakshir, Akbarzad, Batul & Mohaghegh Zade, 2010). Recently this infection has increased dramatically in term of severity and incidence that is mainly due to the increase in cases of immunodeficiency, such as AIDS, cancer, chemotherapy and replacing members (Behmanesh, Pasha, Sefidgar, Moqadam Nia, & Ebrahimi, 2010). It is estimated that 75% of women experience the infection at least once in their lifetime, 40% -50% of patients with recurrence ofVulvovaginitisrefereed to clinics and about 5-8% of infections are frequent in their lifetime. Candida albicans is responsible for 80-95% of VulvovaginitisCandidalattacks around the world.Some research have reported the increased prevalence of other Candida species, especially C. glabrata, krusei and Parapsilosis (Levin et al., 1998; Samaranayake & Samaranayake, 1994; Sobel et al., 2004). Predisposing factors of this disease are including pregnancy, high-dose oral estrogen use, Contraceptives pills, diabetes, broad-spectrum antibiotics use, corticosteroids, immune cell deficiency and obesity. Infection of the vagina or vulva may be associated with symptoms such as severe itching, burning, pain and white or whitish Cottagelike and cheese form secretions (Warren, 2010) The clinical characteristics of patients with vulvovaginal candidacies are nonspecific and their misdiagnosis is common and leads to delays in the initiation of antifungal therapy. However, it is rarely life-threatening infections. Yet it is usually associated with symptoms such as discomfort in sexual disorders, dryness, pain and finally high cost of treatment (Hu et al., 2013; Marot-Leblond et al., 2009). VulvovaginitisCandidal annual cost in the United States of America in 1995 was $ 18 billion and is expected that rise to $ 3.1 billion in 2014 (Foxman, Barlow, D’arcy, Gillespie, & Sobel, 2000). A common treatment for this disease includes high doses of oral or vaginal antifungal azole group followed by weekly or monthly long-term maintenance therapy.Despite the high cost of treatment many women get a little relief from these treatments and some of the women have experienced the side effects of these treatments such as headache, gastrointestinal disorders. The lack of effective treatment result in many women and azole resistance and their consequences and limited number of new antifungal drugs cause high demand to discover new antifungal drugs that are more effective and less toxicity toward available drugs (Sobel et al., 2004; Watson & Calabretto, 2007). Honey as a medicine is being used thousands of years. If it is used alone or in combination with other substances in vaginatoinhibit the growth of Candida (Molan, 1992).

Laboratory and clinical results of honey in inhibiting the growth of various fungi such as Candida has been promising (Devkatte, Zore, & Karuppayil, 2005; Wahdan, 1998). This effect is due to the high acidity and osmolarity (Namias, 2003; Theunissen, Grobler & Gedalia, 2001) as well as hydrogen peroxide, small amounts of enzymes; diastase, invertase, glucose oxidase, Catalase and phosphatase (Allen, Molan, & Reid, 1991).

The existence of herbal derivatives such as flavonoids in honey and stimulating the immune system by stimulating cytogenesis through activating neutrophils and T and B lymphocyteswould lead to increased antibiotic properties of honey (Küçük et al., 2007; Theunissen et al., 2001).

Some studies have investigated the ability of orally administered lactobacilli in the colonization of vaginaorcolonization reduction of vagina with Candida infections (Falagas, Betsi, & Athanasiou, 2006). Lactobacillus and Bifidobacterium have inhibitory effect on many microorganisms through producing lactic and acetic acids bacteriocin, hydrogen peroxide, di-acetyl, acetaldehyde and ammonia (Bromberg, Moreno, Zaganini, Delboni & Oliveira, 2004). Yogurt and Probiotics are commonly used by women when they are infected with vaginal infection and can be used as a cheap and effective treatment for vaginal Vulvovaginal candidiasis. If the lactobacilli in the yogurt directly applied in the vagina can inhibit the growth of fungi in infected women (Ehrström et al., 2010; Falagas et al., 2006). Adding the yogurt and honey increased their antifungal effect and make them to inhibit the growth of Candida Albicans.Other studies also support the synergy of honey in combination with other drugs to prevent candida growth (Boukraâ & Bouchegrane, 2007). Sissons and et al 2004 have reported that Monica honey may be useful in oral fungal infections caused by Candida albicans (Sissons, Anderson, & Molan, 2004). Also Irish and et al(2006) in their study have shown the antifungal effect of honey onCandida albicans, C. glabrata and C. dubliniensis (Irish, Carter, Shokohi & Blair, 2006). Several studies have indicated the effect of yogurt or foreign species of lactobacillus on improvement ofvaginitis candida symptoms vaginitis candida (Osset, Garcia, Bartolome & Andreu, 2001).

Haihong and et al. (2013) in their study have shown the antifungal effect of yogurt (Hu et al., 2013). While few studies do not support the effects of yogurt and probiotics in the prevention of candida (Pirotta, Gunn, & Chondros, 2003; Shalev, Battino, Weiner, Colodner & Keness, 1996). Therefore, considering high incidence of vaginitis and its complications and recurrence of fungal vaginitis despite the increasing of current treatments and the increasing willingness of people to drugs based on natural materials, the present study conducted with aim to investigate the effect of a mixture of yogurt and honey and honey alone on vaginitis candida.

## 2. Method

This study was a randomized, triple blind clinical trial with a control group is registered with number of IRCT201401052248N14. In order to compare the effect of vaginal cream mixture of yogurt and honey on symptoms of Vulvovaginal candidiasis, the study was conducted in clinics and health centers in the city of Sari, Mazandaran University of Medical Sciences in 2013-2014.

The sample size for comparison of two groups determined 70 subjects with confidence level of 95%.

The criteria to enter the study were including; being at age of 18 to 45 years old, Signs and symptoms of vaginal candidiasis in interviews, observation and beingconfirmed by laboratory studies, not being pregnant, no previous use of antifungal drugs, lack of use of broad-spectrum antibiotics and corticosteroids during the last two weeks, the lack of allergy to honey and being non- diabetic, Exclusion criteria included pregnancy, menstruation and abnormal uterine bleeding during treatment. Data collection tool in this study was a questionnaire on demographic variables and confounding background consists of 4 parts 1- (first name, last name, age, BMI, education, job, spouse`s education, spouse`s job, family income in month, housing) 2-Information about menstruation and pregnancy include (number of pregnancy, number of delivery, menstrual pattern, method of contraception) 3- Patient’s history of drug use, including (history of disease, history of drug use, drug allergy history, history of antibiotic use during the past two weeks, the use of yoghurt and honey in meals) 4- Health information includes (Use sanitary pads, cloth napkins, tampons, pool, bath, public bath, a Jacuzzi, a stretch underwear, cotton or synthetic under wear, the use of tight underwear, drying after purification, the average number of times per week having intercourse) B- Assessment of patient complaints check list, Clinical observations, recording the first, second and third reference results including; patient complaints (5 questions), with the option of have answer, no answer, clinical observation consists of 4 questions with options of have answer, no answer, record the results of microscopic evaluation, Culture result with a negative or positive answer, C- Microscope Olympus corporation Tokyo-japan model cx31 Sn-5mo9248, measuring PH Chinese Paper Q/CHSC1544-1999, medium CHROMagar, Paris, France brand, simply slide the glass tube 0 16 * 160.

Methods In this case the researcher after receiving permission from the Ethics Committee of the University of Medical Sciences, Tehran and Mazandaran, in order to collect data referred to the study environment and after explaining the purpose of the study and informed consent was obtained from samples at first History of patients was obtained and in case of having the signs of Vulvovaginal candidiasis (itching, secretionpain during intercourse and dysuria) preliminary questionnaire was completed. In case of no prohibition against the inclusion criteria, the study was fully explained to the patient. Then, with filling the written consent of the study, in order to study the subject was placed in lithotomy position and sterile speculum placed into the vagina without being impregnated material and cervix and vagina were evaluated in terms of symptoms and cheesy white secretions, erythema and even redness, sores and lesions, papulopustular damagesand any abnormal findings. And it was recorded in the observations check list. Then with two sterile cotton swabs (put in an autoclave for 15 minutes at 120 degrees) of vaginal swab samples were taken at the top and side walls secretions.

The first swab was drawn on two slides, added 2 drops of normal saline on first slide and examined under the microscope for the presence of key cells and Trichomonas vaginalis and in case of observing the key cells of Bacterial vaginosis the sample is excluded from the study. 1 drop of KOH 10% solution was added to the second slide and examined under the microscope. If Mycelium and blastorare observed smear test was considered positive. If this test is positive and the second swab placed into the tube containing the normal Saline to transfer to the Chromagar culture medium and sent to laboratory for final diagnosis. Vaginal PH measured by PH meter paper. Secretions PH above the 4.5 suggest mixed infection and were excluded from the study. In this environment, certain colors that are caused by Candida species were determined (Sivakumar, Shankar, Nalina & Menon, 2009). Samples were cultured on Chromagar medium at 37-30 ° C for 72 to 48 hours were placed in the incubator and formed colonies on the medium after 72 hours by the doctor were evaluated macroscopically

Positive culture result makes the diagnosis sure and all Candida species were entered to the study. Then Patients were randomly placed in n a vaginal cream, yoghurt, honey and control groups (clotrimazole vaginal cream). Vaginal cream yogurt and honey including (Solids, insoluble in water: 0.4%, Humidity: 18%, low-fat yogurt, 3.2% fat, dry matter content: 3.7% and pH = 4) by a Pharmacognosy doctor in Armaghan Teb laboratory prepared and packaged in Tehran.

Clotrimazole vaginal cream 1% made by Raha Company with registration number 1228101540 and packaged in the Armaghan Teb laboratory similar to vaginal cream yogurt and honey. And in form of Codes A and B were taken to the researcher. The researcher after reference of samples, a box containing 70 the same cards which was written by an equal number of letters A and B given to the samples and each patient choose a card randomly and the package in the form of written letters on the pharmaceutical form of a vaginal cream was placed in the sample, creams applied each night as an applicator (5 g) inside the vagina for 7 nights. Samples, research group, and statistical analysis were unaware of the content of cream. Therefore, the present study was conducted as a Triple-blind study. All samples were given the guidelines of drug use and health advice including; Daily change of underwear, drying underwear under the sun or ironingit after wash, rinse and dry the genital area from front to back after a bowel movement every time, Use loose cotton underwear. In addition to explain the Educational pamphlet containing health advice that the researcher number is mentioned by the researcher set and is given to the subjects to study at home.

The criteria for exclusion of the study were Pregnancies during the study, unwillingness of subject to continue participation in the study, necessity to use antibiotics during the study, the incidence of allergies to medicines, Failure to observe the correct way to treat (forgetting the drug for more than one night).

Samples were asked to refer to clinic at 7 and 14 days after completion of treatment with a coded card. Also through phone calls the time to visit is reminded to patients again. And the effect of drug therapy on the symptoms of the disease was studied. And it was recorded in observation checklist. The samples were evaluated microscopically. For final confirmation of the presence or absence of fungal, Subculture of samples was prepared. Data Analysis using the chi-square test, McNemar test, ANOVA and Student t using SPSS statistical software (21) was analyzed and P< 0.05 considered as significant level.

**Figure 1:**
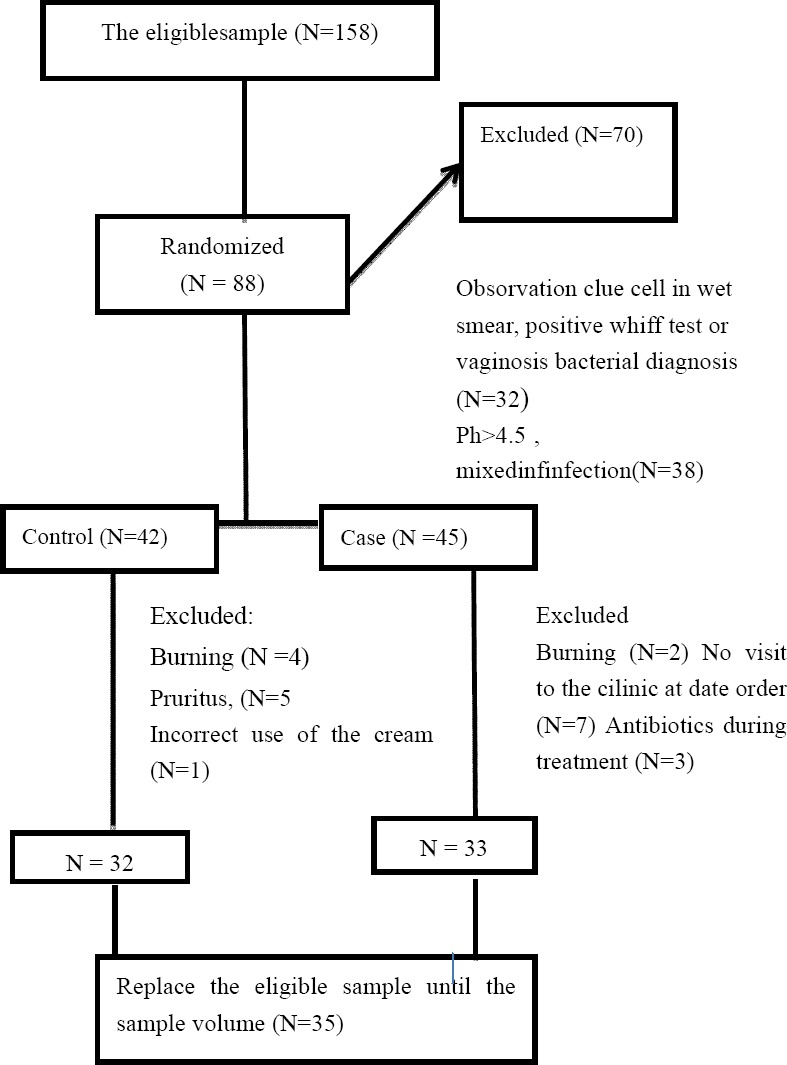
Study procedure diagram

## 3. Results

There was no significant difference between two groups in term of Age average, contraception method, the number of pregnancy and the number of giving birth. (p>0.05) (Table 1).

**Table 1:** Characteristics of women in terms of intervention and treatment groups’variables

	Age	Gravid	Para
Case	32.51±7.54	1.83±1.294	1.57±1.335
Control	33.09±7.64	1.40±1.193	1.23±1.90
P-Value	0.754	0.154	0.261

In Yogurt and honey agroup 20 of subjects (57.1) and in the control group, 20 (57.1) had a regular menstrual cycle. And 12 subjects (34.3) in yogurt and honey, and 11 subjects (31.4) in the clotrimazole group did not use any method of contraception. Chi-square test did not show a significant difference between the two groups in term of menstrual status and contraceptive methods (P > 0.05).

The main complaint at the time of reference was itching atthe “Group of yogurt and honey” 30 subjects (85.7%) and 32 subjects of controls (91.4%). According to the chi-square difference between the two groups was not statistically significant (P>0.05). Symptoms between the two groups before and 7 and 14 days after the intervention are presented in Table 2 and 3. 5.7% in Yogurt and honey group and 28.6% in the clotrimazole group have mentioned the lack of improvement in term of itches in 7 days after the intervention. While this level of lack of improvement of abnormal secretions in yogurt and honey group reported 5.7% andclotrimazole group was 22.9%.

**Table 2:** Distribution of absolute and relative frequency of research units based on the symptoms, treatment phases and groups 7 days after the intervention

	Before treatment			7 day after treatment		
	
	Case	Control	p-value	Case	Control	p-value
Itching	30(85.7)	32(91.4)	0.71	2(5.7)	10(28.6)	0.02
Irritation	27(77.1)	24(68.6)	0.59	2(5.7)	9(25.7)	0.04
Dysuria	18(51.8)	20(57.6)	0.81	1(2.9)	4(11.4)	0.35
Dyspareunia	25(71.4)	22(62.9)	0.61	6(17.1)	9(25.7)	0.59
Discharge	26(74.3)	29(82.9)	0.56	2(5.7)	8(22.9)	0.04

**Table 3:** Distribution of absolute and relative frequency of research units based on the symptoms, treatment phases and groups14 days after the intervention

	Before treatment			14 days after treatment		
	
	Case	Control	p-value	Case	Control	p-value
Itching	30(85.7)	32(91.4)	0.71	1(2-9)	9(25.7)	0.02
Irritation	27(77.1)	24(68.6)	0.59	1(2.9)	8(22.9)	0.04
Dysuria	18(51.8)	20(57.6)	0.81	0(0)	3(8.6)	0.35
Dyspareunia	25(71.4)	22(62.9)	0.61	4(11.4)	8(22.9)	0.59
Discharge	26(74.3)	29(82.9)	0.56	1(2.9)	8(22.9)	0.04

The most symptoms of this infection on clinical examination in two treatment groups, sticky cheesy white secretions in “Group of yogurt and honey” was 30 cases (85.1%) and in the clotrimazole group, was 29 subjects (82.9%).

Based on Fisher’s exact test, there were no significant differences between groups before the treatment of the symptoms. (P > 0.05)

The results showed that after treatment, improvement in symptoms of sticky white cheese like secretionsin Yogurt and honey group was 33 (94.3%) and in the control group was 26 (25.7%). Based on chi-square test between the groups after the treatment of signs the statistically significant difference was observed (P<O.O5). Inflammation and redness of the vulva and vagina before treatment in group of “yogurt, honey,” was 25 (71.4) and in the control group, was 25 (71.4).

Based on chi-square test between the groups before and after treatment of these symptoms, there was no significant difference. (P > 0.05)

After treatment, the improvement of Symptoms of inflammation and redness of the vulva and vagina in Yogurt and honey group 34 subjects (97.1) and in the control group, 27 patients (77.1) was reported. Based on chi-square test, no statistically significant difference between the groups after the treatment of the symptoms was observed (P < 0.05). The other symptoms (pustular lesions and cervical lesions and abnormal cervixhad similar improvement in both groups.Chi-square test did not show statistically significant difference between groups in terms of sings. (P > 0.05)

Present study in both treatment groups, “mixed with yogurt and honey” and clotrimazole showed significant reduction in negative cultures or improvement.

Microscopic evaluation results (smear wet slide) indicated that yogurt and honey 68/6% and clotrimazole group 80% had negative cultures after treatment.

Chi-square test showed a significant difference in terms of reduction of the positive results of the microscopic and culture than before treatment in both groups. (P < 0.05)

In other words, both groups disease improved in terms of the results of microscopy and culture fluids (Table 4).

**Table 4:** Distribution of sample based on microscopic evaluation result of wet slide and fluid culture results, treatment phases and groups

P	Culture	P	Microscopic Evalution	
*<0.001	35(100%)	<0.001	35(100%)	Before treatment
Case				
**NS	24(68.6%)	NS	28(80%)	7 day After treatment
***<0.001	25(71.4%)	<0.001	28(80%)	14 day After treatment

*<0.001	35(100%)	<0.001	35(100%)	Before treatment
control				
**NS	28(80%)	NS	32(91.%4)	7 day After treatment
***<0.001	30(85.7%)	<0.001	32(91.4%)	14 day After treatment

* Before & 7 day aftertreatment;

** 7&14 day aftertreatment;

*** Before & 14 day aftertreatment.

## 4. Discussion

Vulvovaginal candidiasis is the most common clinical protests of Candida species that 70-75% women will experience it at least once in a lifetime. The study is conducted in order to compare the effect of vaginal cream mixed of yogurt and honey on the symptoms of Vulvovaginal candidiasis. The obtained results indicate that signs and symptoms of vaginal candidiasis after taking 7 day a vaginal cream yogurt and honey mixed is reduced significantly. Results of the study showed that recovery in term of itches in 7 days after treating with mixed of yogurt and honey happened in more than ¾ samples and in clotrimazole group occurred in almost two-thirds of the cases. In this regard, the result of study of Fazel and et al have reported the Itching recovery in the honey group alone and in combination with clotrimazole was 100% and clotrimazole group less than a quarter of the samples which demonstrated the alignment with present study. The difference between the symptoms of the current study may be related to the use of different measuring instruments (Fazel, Hashemian, Ramezani & Akaberi, 2011).

In addition to study results of Allam and et al n 129 pregnant women with Vulvovaginitiscandidiasissuggests that the use of yogurt and honey mixture in comparison with itraconazole group (87.8 percent vs. 72.3 percent) improves symptoms of Vulvovaginitiscandidiasis. So that itching, secretions, and redness of the vulva and vagina, has significantly decreased in consumer group of vaginal cream, yogurt and honey compared to itraconazole group (Abdelmonem et al., 2012). In the study of Hilton and et al also the vaginal symptoms of women who had used the Jay intravaginal Lactobacillus for 1 or 2 months have recovered (Hilton, Isenberg, Alperstein, France & Borenstein, 1992). Also the study result of Ehrstrom and et al indicated that the effect of supplements and probiotic lactobacillus capsules in the treatment of bacterial vaginosis and Vulvovaginitiscandidiasis (Ehrström et al., 2010).

It is assumed that antifungal and anti-inflammatory effect of honey and being rich of lactobacilli and lactic acid in yogurt is that make vaginal environment unsuitable for the growth of candidiasis. Also the honey through producing the prostaglandins reduces the edema and pain in the inflammatory tissues. In the present study yogurt and honey mixture cause inhabitation of colonization of Candida species which is consistent with the findings of the study of Irish and et al. (Pirotta et al., 2003). This effect is likely related to the antimicrobial effect of honey is due to its Osmolality.

In the present study, the combination of honey with yogurtcauses a synergistic effect of honey against candidiasis which is consistent with the finding of Boukraaand et al based on the synergistic effect of honey and starch against Candida albicans (Boukraâ & Bouchegrane, 2007).

The present findings Demonstrates the effectiveness of honey and Yogurt on Negative cultures of samples within 7 days after the intervention, while no effect was observed in the study of Shalev and et al. (1996). It is assumed that difference is due to using honey in mixture with Yogurt and the antimicrobial effect of honey is due to its chemical composition and PH of honey is acidic in the present study. Since one of the criteria for inclusion in the study was the lack of use of antibiotics in the sampling, therefore the results of the present study was inconsistent with the study result of Pirotta and et al about the role of oral or vaginal lactobacilli strains in the prevention of Vulvovaginal candidiasis after use of antibiotics.

In summary, the present study showed that prescribing the honey in mixture with yogurt in from of vaginal is effective in reducing symptoms of Vulvovaginal candidiasis And also had a negative impact on the results of microscopy and culture fluids while taking any of these serious side effects have been reported.

The limitations of this study can be mentioned as Differences in immune systems of subjects and relatively short follow-up period. And also Sample loss during the investigation, lack of reference of some subjects for culture after treatment were other limitation of the study which leads to the removal of above mentioned cases and re-sampling and subsequently increasing the research time. The strengths point of present study is having a control group, designing the study in Triple-blind and randomized into two groups of samples.

## 5. Conclusion

In the present study administration of honey in combination with yogurt has desired effect in the treatment of vaginal candidiasis. And cause the reduction and recovery of vaginitis candida. Also it is more economical in terms of costs. And don’t have the synthetic drugs side effects. Therefore, their use in combination with topical formulations for the treatment of vaginal candidiasis wouldbe useful.
